# Triazoles and Their Derivatives: Chemistry, Synthesis, and Therapeutic Applications

**DOI:** 10.3389/fmolb.2022.864286

**Published:** 2022-04-25

**Authors:** Mohammed M. Matin, Priyanka Matin, Md. Rezaur Rahman, Taibi Ben Hadda, Faisal A. Almalki, Shafi Mahmud, Mohammed M. Ghoneim, Maha Alruwaily, Sultan Alshehri

**Affiliations:** ^1^ Bioorganic and Medicinal Chemistry Laboratory, Faculty of Science, Department of Chemistry, University of Chittagong, Hathajari, Chittagong, Bangladesh; ^2^ Department of Chemical Engineering and Energy Sustainability, Faculty of Engineering, Universiti Malaysia Sarawak, Kuching, Malaysia; ^3^ Department of Pharmaceutical Chemistry, Faculty of Pharmacy, Umm Al-Qura University, Makkah, Saudi Arabia; ^4^ Genetic Engineering and Biotechnology, University of Rajshahi, Rajshahi, Bangladesh; ^5^ Department of Pharmacy Practice, College of Pharmacy, AlMaarefa University, Ad Diriyah, Saudi Arabia; ^6^ Department of Pharmaceutics, College of Pharmacy, King Saud University, Riyadh, Saudi Arabia

**Keywords:** anticancer agents, azide–alkyne cycloaddition, cefatrizine, isomeric triazoles, microwave-assisted green synthesis, pharmacological applications, SARS-CoV-2, triazole–thiazole hybrids

## Abstract

Among the nitrogen-containing heterocyclic compounds, triazoles emerge with superior pharmacological applications. Structurally, there are two types of five-membered triazoles: 1,2,3-triazole and 1,2,4-triazole. Due to the structural characteristics, both 1,2,3- and 1,2,4-triazoles are able to accommodate a broad range of substituents (electrophiles and nucleophiles) around the core structures and pave the way for the construction of diverse novel bioactive molecules. Both the triazoles and their derivatives have significant biological properties including antimicrobial, antiviral, antitubercular, anticancer, anticonvulsant, analgesic, antioxidant, anti-inflammatory, and antidepressant activities. These are also important in organocatalysis, agrochemicals, and materials science. Thus, they have a broad range of therapeutic applications with ever-widening future scope across scientific disciplines. However, adverse events such as hepatotoxicity and hormonal problems lead to a careful revision of the azole family to obtain higher efficacy with minimum side effects. This review focuses on the structural features, synthesis, and notable therapeutic applications of triazoles and related compounds.

## Introduction

**FIGURE 1:**
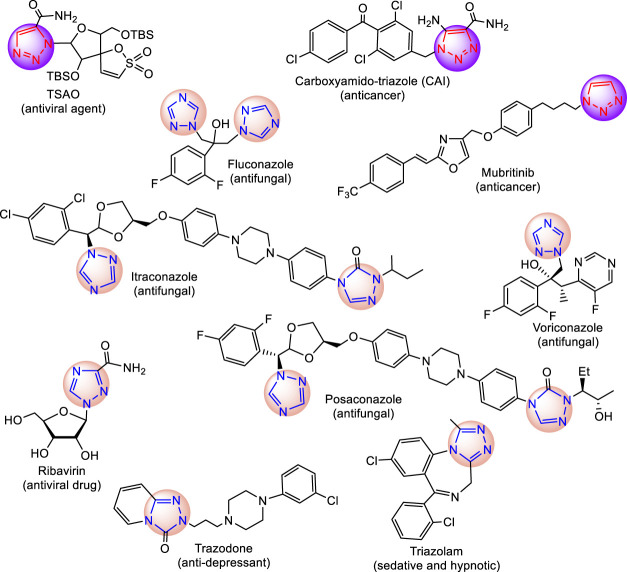
Medicinal drugs with triazole nucleus.

The name triazole was first coined by Bladin in 1885 to assign the five-membered three nitrogen–containing heterocyclic aromatic ring system having molecular formula C_2_H_3_N_3_ (Bladin, 1885). After the discovery of triazole, its chemistry was developed gradually and speeded up with the establishment of several facile and convenient synthetic techniques along with its versatile interaction with biological systems (Aneja et al., 2018; Shafiei et al., 2020; Farooq, 2021). For example, discovery of antifungal activities of azole derivatives in 1944 (Woolley, 1944) led to the invention of fluconazole, itraconazole, voriconazole, posaconazole, efinaconazole, *etc*. (Figure 1; Zonios and Bennett, 2008). Of these, voriconazole and posaconazole are active against fluconazole-resistant strains of *Candida*. The mechanism of such antifungal action is also well-established which involves the inhibition of ergosterol synthesis and blocking of the P450-dependent enzyme (CYP 51) (Odds et al., 2003). Triazole-type ring structure(s) can coordinate with the heme iron of the CYP enzyme (Zhang et al., 2014).

In addition, triazole heterocyclic structures are found to form many weak nonbond interactions with the receptors and enzymes in biological systems (Hitchcock et al., 1990). These inherent properties of triazole compounds have established them as key chromophores with immense medicinal value and attract scientists of all disciplines, including chemical, agricultural, supramolecular, pharmaceutical, polymer, and materials sciences (Chang et al., 2011). Among the medicinal drugs, triazole-based antibacterial, antifungal, antiviral, anti-inflammatory, anticoagulant, antitubercular, antidiabetic, antioxidant, and anticancer drugs are available (Kumar et al., 2021).

The appearance of multidrug-resistant (MDR) pathogens, especially, resistance to triazole drugs makes microbial treatment less effective, a worse prognosis of infection, and problematic (Sagatova et al., 2016). For example, *Candida albicans* and *Candida krusei* strains (responsible for 75–88% of fungal infections) are resistant to the most common azole drug fluconazole (Berkow and Lockhart, 2017). Azole-derived several drugs have also become resistant against *A. fumigatus* and *C. glabrata* strains (Faria-Ramos et al., 2014). In addition, many adverse effects such as rash, diarrhea, headache, hepatotoxicity, and gastrointestinal problems including several severe problems (heart failure, renal failure, liver problems, Stevens–Johnson syndrome, *etc*.) are reported for many triazole drugs (Yang et al., 2021). Thus, the prudential development of new triazole drugs with bioisosteric replacement and molecular hybridization is necessary to overcome MDR pathogens and reduce the side effects of the available drugs. In this review, structural features, synthetic approaches, and biological properties of 1,2,3- and 1,2,4-triazoles are discussed, highlighting the related research works since 2015.

### Chemistry of Triazoles

**FIGURE 2:**
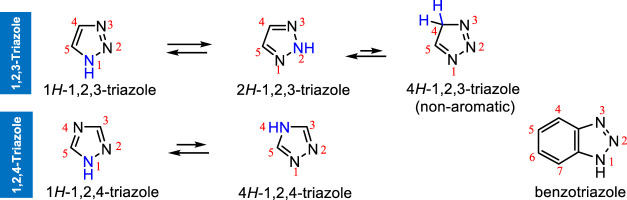
Structures of isomeric triazoles.

Due to a wide range of applications across scientific disciplines, triazoles gained an exceptional structural motif and are notably related to the chemistry of triazoles. The basic skeleton of triazoles comprises a five-membered heterocyclic ring consisting of two carbon and three nitrogen atoms with the molecular formula C_2_H_3_N_3_. In the five-membered ring, a maximum of two types of positional arrangement of nitrogen atoms led to the formation of two substantial isomers, namely, 1,2,3-triazole (ν-triazole) and 1,2,4-triazole (s-triazole). Each of them shows mainly two tautomers depending on the hydrogen bonded to ring nitrogen (Figure 2). The 4*H*-1,2,3-triazole structure is nonaromatic and hence is discarded. All the atoms in both the triazoles are in *sp* ^2^ hybridized and are planar. Six pi (π) electrons are available in both forms, which are delocalized around the ring to generate their aromatic character. Moreover, the presence of 3 N atoms makes triazoles energy-rich heterocycles (Tao et al., 2009; Gao and Shreeve, 2011).

When a benzene ring is fused at the 4,5-positions of 1,2,3-triazoles, it is termed benzotriazoles (Figure 2). In the monocyclic 1,2,3-triazoles, both 1*H*- and 2*H*-1,2,3-triazoles are generally in equilibrium in both solution and gas phases and exist as an equimolar mixture in the solid state. However, in an aqueous solution, 2*H*-1,2,3-triazole exists as major compared to the other tautomer (2*H*:1*H* = 2:1) (Albert and Taylor, 1989). The parent 1*H*-1,2,3-triazole is a clear liquid with a bp of 203°C (Ram et al., 2019), computed topological polar surface area of 41.6 Å^2^, and is soluble in H_2_O. Most of the 1,2,3-triazoles are prepared from azides. The presence of one pyrrole-type and two pyridine-type nitrogen atoms makes 1,2,3-triazole rings very stable and difficult for quaternization. It easily undergoes electrophilic substitution at carbon or at nitrogen.

In 1,2,4-triazoles, the parent 1*H*-1,2,4-triazole is a white powder solid (mp 120–121°C, bp 260°C). Like 1*H*-1,2,3-triazole, it is very soluble in water. It is also soluble in organic solvents. The two tautomers (1*H*- and 4*H*-) of 1,2,4-triazoles are in rapid equilibrium. However, 1*H*-1,2,4-triazole is more stable than the 4*H*-1,2,4-triazole (Potts, 1961). Chemically, 1*H*-1,2,4-triazole shows both electrophilic and nucleophilic substitution reactions. Due to high electron density, electrophilic substitution occurs at nitrogen atoms only. Under mild reaction conditions, nucleophilic substitution occurs at both the ring carbon atoms. This is because both the ring carbon atoms are attached to two electronegative nitrogen atoms and become π-deficient, which makes them susceptible to nucleophiles.

## Synthetic Approaches

Huge applications, promising research directions, and lower molecular toxicity of various triazoles and their derivatives have promoted the researchers to design many synthetic strategies. Availability of reagents and simplicity of synthetic procedures justified the fact.

### 1,2,3-Triazole Analogs

In neoteric chemistry, the 1,2,3-triazole group is one of the most significant functional aromatic heterocyclic systems.

#### Cu(I)-Catalyzed Azide–Alkyne Cycloaddition (CuAAC)

The uncovering of the Cu(I)-catalyzed azide–alkyne cycloaddition (CuAAC) helps the inception of click chemistry and is mainly used as a unique tool to synthesize a wide variety of 1,4-disubstituted triazole compounds (Tornøe et al., 2002; Mao et al., 2020). CuAAC is well known for its inexpensive catalytic systems and generates highly regioselective products (Rostovtsev et al., 2002).

Lebeau et al. (2016) utilized Huisgen 1,3-dipolar cycloaddition of terminal alkynes (**1)** with methyl 2-azidoacetate (**2)** in the presence of Cu(I) and obtained 1,4-disubstituted-1,2,3-triazole derivatives (**3)** in high yields at 25°C (Supplementary Figure S1B). The general Huisgen 1,3-dipolar cycloaddition reaction of azides with alkynes under heating conditions produces an equal mixture of 1,4- and 1,5-disubstituted isomers (Huisgen, 1963). However, the use of Cu(I) catalyst in such a one-pot reaction shows regioselectivity with the formation of only the 1,4-disubstituted isomer and is a model example of click chemistry (Tornøe et al., 2002). Encouragingly, many of the 1,4-disubstituted-1,2,3-triazoles (**3)** showed notable inhibitory activities against Src kinase, and hence could be effective in cancer treatment (Lebeau et al., 2016).

A copper-catalyzed click reaction was used to prepare benzimidazole-linked 1,2,3-triazoles (**6)** (Supplementary Figure S1B). The key step involves the CuAAC reactions between aromatic azides (**4)** and *n*-propynylated benzimidazole (**5)** *via* a copper catalyst (Bakherad et al., 2019). In this method, the ligand is not necessary, and hence, the purification process of this reaction is very simple.

Similarly, 1,3-dipolar cycloaddition of *n*-alkyl propargyl ethers (terminal alkynes) (**7)** with substituted azidoacetamide (**8)** furnished corresponding substituted 1,2,3-triazoles (**9)** (Ibraheem et al., 2019) in good yields (55–81%; Supplementary Figure S1C).

#### Metal-Free Click Synthesis

In 2016, a metal-free three-component new protocol was reported for the direct and selective synthesis of 1,5-disubstituted-triazoles (**13)** (Thomas et al., 2016). In this approach, primary amines (**10)**, enolizable ketones (**11)**, and 4-nitrophenyl azide (**12)** in the acetic acid catalyst (30 mol%) are heated at 100°C in toluene (Supplementary Figure S2A).

A straightforward, metal-free, and expandable click protocol for the preparation of 1-substituted-1,2,3-triazoles is reported (Giel et al., 2020). They used ethenesulfonyl fluoride (ESF) as a stable, the most perfect Michael acceptor, and an efficient acetylene surrogate. Thus, treatment of azide (**14**) with ESF in EtOAc under reflux condition furnished 1-substituted-1,2,3-triazole(s) (**15**) in good-to-excellent yield (Supplementary Figure S2B; Giel et al., 2020). However, the performance of the reaction at ambient temperature in benzene is unsuccessful (Rondestvedt and Chang, 1955).

This approach is suitable for the synthesis of many drug and drug fragments with 1-substituted-1,2,3-triazole (e.g., chloramphenazole, triazolyl oseltamvir, triazolyl dapsone, *etc*.). In addition, a similar strategy is useful for the synthesis of 1-substituted-1*H*-1,2,3-triazole-4-sulfonyl fluorides (**15a**) by changing ESF to BESF (1-bromoethene-1-sulfonyl fluoride) (Smedley et al., 2018; Thomas and Fokin, 2018).

#### Organocatalytic 1,3-Dipolar Cycloaddition Reaction

Over the last decades, organocatalytic azide-carbonyl [3 + 2]-cycloaddition (OrgACC) reactions received significant attention. The versatility of such reactions is also applied in the synthesis of 1,2,3-triazoles *via* enamine/enolate-mediated organocatalysis (Tsogoeva et al., 2017). In this regard, a series of aliphatic and cyclic tertiary amines are extensively investigated, and 1,8-diazabicyclo [5.4.0]undec-7-ene (DBU) was established as the most effective catalyst (Zhou et al., 2016). Among different solvents, DMSO and chloroform are found as the best solvent for this DBU-catalyzed 1,3-dipolar cycloaddition reaction.

In 2015, a group of researchers (Li et al., 2015) developed the application of DBU as a catalyst in organocatalytic [3 + 2] 1,3-dipolar cycloaddition between α,β-unsaturated esters and azides. This synthetic strategy is found to form regioselective 1,4-disubstituted-1,2,3-triazoles (**16)** in high yields. A highly important class of triazoles are found to be 1,4-disubstituted-1,2,3-triazoles. For example, ammonolysis of such triazole (**16a)** can form pharmaceutically important agents such as rufinamide (**17**; Supplementary Figure S2C).

Later on, the same group (Zhou et al., 2016) successfully extended the aforementioned organocatalytic (DBU) strategy with β-keto amides (**18)** and obtained highly substituted 1,2,3-triazole-4-carboxamides (**19a-z)** in excellent yields with substituent regioselectivity at 1-, 4-, and 5-positions (Supplementary Figure S2D). They have also outlined the probable mechanism.

Application of a similar strategy by the change of azide to imidazole sulfonyl azide with β-keto esters provides an efficient one-pot practical method for *N*-amino-triazole synthesis (Nagarajan et al., 2016). As shown in Supplementary Figure S3A, hydrazine (**21**) was obtained from β-keto esters (**20**), which on treatment with imidazole sulfonyl azide catalyzed by DBU-furnished 1,2,3-triazoles (**22)** containing nitrogen atom at the 1-position. This simple protocol is found to be applicable to acyclic and cyclic 1,3-diones. In addition, the extra NH group at 1-position can form H-bond with many biological systems.

Finally, the metal-free OrgACC reaction promoted by DBU in DMSO is found suitable for the preparation of functionally rich vinyl-/alkyl-/aryl-containing 1,2,3-triazoles under ambient conditions (Reddy et al., 2020). It should be noted that the less reactive vinyl/alkyl/aryl azides could be successfully used in these reaction conditions. For example, cyclic enone (**23**) with vinyl azide (**24**; α-azidostyrene) catalyzed by DBU furnished the corresponding *C*/*N*-divinyl-1,2,3-triazole (**25**) (Supplementary Figure S3B). These *C*-vinyl- and *N*-vinyl-triazoles have many biological activities, including EP4 receptor antagonists, α-glycosidase inhibition, antitubercular, antimicrobial, tubulin inhibition, and anti-inflammatory properties (Yang et al., 2020).

#### Ionic Liquid–Catalyzed Synthesis

Since 2015, several researchers reported the applicability of ionic liquids (ILs) as a solvent and catalyst for the regioselective synthesis of 1,2,3-triazoles. The use of ILs as nontoxic benign solvents can improve the reaction rate and regioselectivity of the cycloaddition reaction (Javaherian et al., 2014). A simple bifunctional IL catalyst, namely, choline chloride-CuCl was found highly active for the [3 + 2] Huisgen cycloaddition in H_2_O (Liu et al., 2016). In a one-pot, three-component reaction among organic halide (**26)**, NaN_3,_ and terminal alkyne (**27)** with this IL catalyst formed 1,4-disubstituted-triazole (**28**) (Supplementary Figure S3C).

In 2018, another group of researchers reported the application of 1-methyl pyridinium trifluoromethane sulfonate ([mPy]OTf) as an efficient and reusable homogenous IL catalyst in the eliminative azide–olefin cycloaddition (EAOC) reaction (De Nino et al., 2018). Thus, the reaction between substituted azides (**29)** and nitroolefins (**30a-n)** catalyzed by [mPy]OTf/FeCl_3_ yielded 1,5-disubstituted-1,2,3-triazoles (**31a-n)** (Supplementary Figure S3D). The reaction proceeded in a very short reaction time with higher regioselectivity and the final products.

Later on, the same authors applied this IL [mPy]OTf with H_2_O/Er(OTf)_3_, which matches with the anionic part of the IL and produces similar 1,5-disubstituted-triazoles (Maiuolo et al., 2019). This catalyst system can be reused five times with a simple work-up procedure.

In the next year, the same research group developed an IL-catalyzed novel synthetic route with 1,3-dipolar cycloaddition (Huisgen’s-concerted asynchronous) followed by base-promoted elimination (retro-aza-Michael) for the preparation of trisubstituted triazoles from aryl azides and enaminones (De Nino et al., 2019). According to this strategy, stirring of a mixture of enaminone (**32a-c)** and azide (**33a-g)** (2 eq) with [mPy]OTf -water (5:0.5 v/v) and Et_3_N (2 eq) at 100°C formed the trisubstituted products (**34a-p)** with the regioselectivity at 1-, 4-, and 5-positions (Supplementary Figure S3E). Thus, this IL-promoted regioselectivity is different from the previously mentioned ionic liquid/iron (III) chloride method, which showed regioselectivity at 1- and 5-positions of the 1,2,3-triazole skeleton (De Nino et al., 2018). For an in-depth understanding of the role of catalysts, a detailed comparison of different ILs and other catalysts used is also outlined (De Nino et al., 2021).

Recently, Cu(II) IL ([Bmim][CuCl_3_])–promoted regioselective preparation of 1,4-disubstituted-1,2,3-triazole is reported (Phukan et al., 2021). The necessary IL [Bmim][CuCl_3_] is prepared from CuCl_2_ and 1-butyl-3-methylimidazolium chloride. The reaction of azide (**35)** with 1-alkyne (**36)** in the presence of catalyst [Bmim][CuCl_3_] and reducing agent ascorbic acid at room temperature yielded triazole (**37)** (Supplementary Figure S4A). Here, *in situ* generations of the active Cu(I)-IL from Cu(II)-IL by ascorbic acid advantageously facilitate the rapid formation of the product.

#### Microwave-Assisted Synthesis

Recently (Joy et al., 2020), microwave irradiation (MWI) was applied for the copper-catalyzed azide–alkyne cycloaddition (CuAAC) method. Thus, MWI of initially prepared 4-methyl-7-propargylated coumarin (terminal alkyne) (**38)** with various substituted azides (**39)** in sodium ascorbate and hydrated copper sulfate (CuSO_4_.5H_2_O) at 90°C undergoes 1,3-dipolar cycloaddition reaction and furnished 1,2,3-triazoles (**40a-t)** linked with coumarin at the C-4 position in 2–5 min with 97% isolated yield (Supplementary Figure S4B).

Coumarin triazoles (**40a-t)** exhibited promising antibacterial activity compared to the standard drug, ciprofloxacin, and fungal pathogens (Joy et al., 2020). This observation is also supported by a higher binding affinity of (**40)** (−6.3 to −7.2 kcal/mol) than that of ciprofloxacin (−6.2 kcal/mol) with the gyrase enzyme.

The change of CuSO_4_.5H_2_O to Zn(OAc)_2_ and H_2_O in the earlier MW-assisted CuAAC reaction also proceeds with similar regioselectivity (1,4-disubstituted products) and is considered an environmentally friendly inexpensive catalyst in neat water (Morozova et al., 2017).

#### f) Miscellaneous Methods

A new and efficient catalyst-like monophosphine Cu(I) complex containing bis(pyrazolyl)methane (L_1_) (CuIL_1_PPh_3_) under ultrasonic (US) conditions was used in the three-component click reaction, and disubstituted 1,2,3-triazoles are formed (Castillo et al., 2020). Thus, CuIL_1_PPh_3_ catalyzed a one-pot reaction of alkyl halide (**41)**, sodium azide, and terminal alkyne (**42)** under US conditions in water-furnished 1,4-disubstituted-triazoles (**43)** (Supplementary Figure S4C). This CuIL_1_PPh_3_ catalyst is compatible with oxygen/water and triazoles (**43a-l)**, which are formed in a shorter reaction time. The halo-aryl–substituted products are modified *via* Suzuki–Miyaura cross-coupling to add pharmacophore(s), which exhibited better binding affinity with the carbonic anhydrase-II enzyme (Avula et al., 2021).

### 1,2,4-Triazole Analogs

Due to the role as key skeletons of a plethora of biologically active molecules, several synthetic methods for 1,2,4-triazole synthesis were reported since 2015 along with many reviews. Some of the synthetic strategies such as Cu-catalyzed synthesis, base-catalyzed synthesis, MW-assisted methods, and miscellaneous methods are highlighted here based on synthetic convenience, diversity, novelty, and good yields (more synthetic 1,2,4-triazoles are mentioned in the Supplementary Material).

#### Cu-catalyzed Synthesis

In 2015, Cu catalyzed an efficient one-pot synthetic strategy described for symmetrically and unsymmetrically substituted 1,2,4-triazoles from hydroxylamine and nitriles in moderate yields (Xu et al., 2015). The strategy consists of intermolecular addition of hydroxylamine to the first nitrile to provide amidoxime followed by the reaction of the second nitrile in the presence of Cu and intramolecular cyclization to yield disubstituted triazole (**44)** (Supplementary Figure S5A). In the second step, sequential N–C and N–N bond formation occurs by dehydration.

#### Base-Catalyzed Synthesis

Base-catalyzed (ethanolic NaOH/KOH/NaHCO_3_) cyclization of acyl thiosemicarbazide (**45a-c)** under reflux condition paves easy access to the 3-aryl-5-mercapto-1,2,4-triazoles (**46a-c)** (Supplementary Figure S5B; Mioc et al., 2017). Triazoles (**46a-c)** exhibited *in vitro* anticarcinogenic susceptibility against the breast cancer cell line (MDA-MB-231) (Mioc et al., 2017). In addition, 5-mercapto-1,2,4-triazoles (**46a-c)** can be used as a scaffold for the preparation of *S*-substituted triazoles with antiproliferative activities in colorectal cancer (Mioc et al., 2018).

Similarly, 5-mercapto-1,2,4-triazole with 4-amino skeleton (**47)** is shown to be extremely useful for the synthesis of fused triazolo–trizines **48** (El-Reedy and Soliman, 2020; Supplementary Figure S5C). These compounds showed excellent antimicrobial and anti-inflammatory activities compared to commercial antibiotics.

#### MW-Assisted Method

Microwave (MW) heating was used for condensation between *t*-butyl-1-cyanopiperazine carboxylate (**49)** and 2-fluorobenzohydrazide (**50)** in DMF at 120°C to produce the corresponding 3,5-disubstituted-1,2,4-triazole-based piperazine (**51)** (Supplementary Figure S6A). The cyclization proceeds with a high yield (>99%) and did not use any base. Triazole (**51)** was also converted into several amides and urea derivatives mostly under mild MW conditions (Vaithiyalingam et al., 2021).

#### Miscellaneous Method

New spiro-type 1,2,4-triazoles (**53)** are prepared successfully from amidrazones (**52)** with cyclic ketones using *p*-toluenesulfonic acid (*p*-TSA) as the catalyst (Dalloul et al., 2017; Supplementary Figure S6B). These spiro-triazoles possess marked antimicrobial activities and are comparable to tetracycline and fluconazole.

1,2,4-triazoles (**54a-m)** with pyrazole and thioether moieties (3,5-disubstituted) are reported (Zhai et al., 2017). The synthesis was accomplished within six steps (Supplementary Figure S6C). Encouragingly, compounds (**54a-m)** exhibited fungicidal and herbicidal activities.

In 2021, a multistep synthetic route for 2,3,4-trisubstituted-1,2,4-triazoles (**56)** was reported, where the key step involves the replacement of the ring oxygen of oxadiazole (**55)** by the addition of hydrazine hydrate (Supplementary Figure S6D). The biological screening and SAR of these trisubstituted derivatives indicated promising antimicrobial and anticancer activity against HCT116 cell lines (Kumari et al., 2021). For anticarcinogenic studies, the *N*-amino-1,2,4-triazole-type compounds (**56)** are also converted into several new Schiff bases (**57)** (Abdulghani et al., 2022).

## Therapeutic Application

The presence of the three nitrogen atoms in triazole structures afforded opportunities for a plethora of structural modification with the generation of novel therapeutically potential agents, which is different from other heterocyclic compounds (Dhavale and Matin, 2004; Matin et al., 2005). Thus, triazoles are a significant platform in medicinal chemistry and chemical biology, which play key roles in various biological mechanisms related to infections, cancer, convulsions, inflammation, neurodegeneration, and oxidative stress (Hahm et al., 2020; Kumar et al., 2021). Relatedly, many drugs are available in the market. However, the synthesis of newer triazoles is in a continuous process for uncovering unexplored and advanced pharmacological implications.

Bioactive molecules with 1,2,3-triazole core nucleus have been proven to possess antibacterial (e.g., cefatrizine, Supplementary Figure S7), antifungal, herbicide, anticancer (e.g. carboxyamidotriazole or CAI) protease inhibitory, and antituberculosis activities (Zhou et al., 2016; Celik et al., 2018). In search of novel modes of action, many novel 1,2,3-triazoles have been synthesized since 2015. As an instance, a group of researchers discovered that icotinib-1,2,3-triazole derivatives (**58)** (Supplementary Figure S7) exhibited remarkable inhibitory activity against indoleamine 2,3-dioxygenase 1 (IDO1) with very low IC_50_ values (0.37–2.50 μM), and hence are potential anticancer agents (Mao et al., 2020). These IDO1 inhibitors form a coordinate bond with the heme iron of IDO1.

Triazoles linking nonsteroidal anti-inflammatory drugs (NSAIDs) and heterocyclic moiety such as **59** showed excellent inhibition against Gram-negative *P. aeruginosa* with anticancer properties (COLO-205 and HOP-62 cell-lines) (Kuntala et al., 2021). Again, 1,2,3-triazoles (**60)** with short nonpolar alkyl or alkynyl substituents at 1,4 positions showed promising soil nitrification inhibition (pH 7.3) (Taggert et al., 2021). These triazoles can keep the effectiveness of existing nitrogen fertilizers by inhibiting nitrification, especially at elevated soil temperatures.

In the last couple of years, several substituted 1,2,3-triazoles were investigated for their efficacy against severe acute respiratory syndrome coronavirus 2 (SARS-CoV-2), and most of them are based on *in silico* analyses. For example, combined 1,2,3-triazole and tetrazole moieties as in (**61)** (Supplementary Figure S7) are found to inhibit the main protease (M^PRO^, PDB ID: 6LU7) of SARS-CoV-2 having higher ligand–target interactions (Cortés-García et al., 2020). Several functionalized 1,2,3-triazole derivatives (**62)** also showed good binding affinities (−6.0 to −8.8 kcal/mol) against the same protease 6LU7 (Aouad et al., 2021), and 1,2,3-triazoles (**63)** conjugated with quinolone also showed high potency against M^PRO^ 6LU7 (Seliem et al., 2021). The antiviral results of (**63)** are also supported by their IC_50_ values (0.060–0.204 mM).

1,2,3-triazole–based Schiff bases **64** (Supplementary Figure S7) showed considerable binding affinities (−7.4 to −9.1 kcal/mol) with 7BQY, indicating their potential prospect as therapeutics for COVID-19 (Supplementary Figure S7) (Said et al., 2021).

Many 1,2,4-triazole-derived drugs are used as antifungal, herbicidal, antiviral, and catalase inhibitors. Very recently, mefentrifluconazole was introduced to the European market as an effective fungicide (Jørgensen et al., 2021). Several 1,2,4-triazoles such as (**65a-b)** (Supplementary Figure S7) are potent against Middle East respiratory syndrome coronavirus (MERS-CoV) helicase. The experimental (IC_50_ = 8.9–12.4 μM L^−1^) and *in silico* docking study indicated nsp13 as the most active binding site (Zaher et al., 2020).

For more information about the biological significance of triazoles, please refer to the Supplementary File.

## Conclusion

The core triazole ring structures with higher aromatic stabilization energy are modified for improving solubility and selectivity with the interacting binding site of the enzyme and acted as linkers among various pharmacophores. Thus, they have been shown to play a vital role in a wide range of biological activities, including fragment-based drug design, biomolecular mimetics, and bioorthogonal methodologies. In addition to the available triazole drugs, researchers are engaged to explore and develop new scaffolds based on triazole cores with huge applications in biomedical and biotechnology fields. In the present review, structural features, recent synthetic developments, and new biological applications of triazoles are highlighted, which might facilitate in-depth understanding and further development/discovery of these compounds.

## References

[B1] Abdulghani S. M. Al-Rawi M. S. Tomma J. H. (2022). Synthesis of New 1,2,4-triazole Derivatives with Expected Biological Activities. Chem. Methodol. 6 (1), 59–66. 10.22034/chemm.2022.1.6

[B2] Albert A. Taylor P. J. (1989). The Tautomerism of 1,2,3-triazole in Aqueous Solution. J. Chem. Soc. Perkin Trans. 2 2, 1903–1905. 10.1039/P29890001903

[B3] Aneja B. Azam M. Alam S. Perwez A. Maguire R. Yadava U. (2018). Natural Product-Based 1,2,3-triazole/sulfonate Analogues as Potential Chemotherapeutic Agents for Bacterial Infections. ACS Omega 3, 6912–6930. 10.1021/acsomega.8b00582 30023966 PMC6044994

[B4] Aouad M. R. Khan D. J. O. Said M. A. Al‐Kaff N. S. Rezki N. Ali A. A. (2021). Novel 1,2,3‐Triazole Derivatives as Potential Inhibitors against Covid‐19 Main Protease: Synthesis, Characterization, Molecular Docking and DFT Studies. ChemistrySelect 6, 3468–3486. 10.1002/slct.202100522 34230893 PMC8250976

[B5] Avula S. K. Khan M. Halim S. A. Khan A. Al-Riyami S. A. Csuk R. (2021). Synthesis of New 1H-1,2,3-Triazole Analogs in Aqueous Medium via "Click" Chemistry: A Novel Class of Potential Carbonic Anhydrase-II Inhibitors. Front. Chem. 9, 642614. 10.3389/fchem.2021.642614 34277561 PMC8278147

[B6] Bakherad M. Keivanloo A. Amin A. H. Farkhondeh A. (2019). Synthesis of 1,2,3 Triazole-Linked Benzimidazole through a Copper-Catalyzed Click Reaction. Heterocycl. Commun. 25, 122–129. 10.1515/hc-2019-0016

[B7] Berkow E. Lockhart S. (2017). Fluconazole Resistance in Candida Species: a Current Perspective. Idr 10, 237–245. 10.2147/IDR.S118892 PMC5546770 28814889

[B8] Bladin J. A. (1885). Ueber von dicyanphenylhydrazin abgeleitete verbindungen. Ber. Dtsch. Chem. Ges. 18, 1544–1551. 10.1002/cber.188501801335

[B9] Castillo J.-C. Bravo N.-F. Tamayo L.-V. Mestizo P.-D. Hurtado J. Macías M. (2020). Water-compatible Synthesis of 1,2,3-triazoles under Ultrasonic Conditions by a Cu(I) Complex-Mediated Click Reaction. ACS Omega 5 (46), 30148–30159. 10.1021/acsomega.0c04592 33251449 PMC7689893

[B10] Celik F. Unver Y. Barut B. Ozel A. Sancak K. (2018). Synthesis, Characterization and Biological Activities of New Symmetric Bis-1,2,3-Triazoles with Click Chemistry. Mc 14 (3), 230–241. 10.2174/1573406413666171120165226 29165092

[B11] Chang J. -J. Wang Y. Zhang H. -Z. Zhou C. -H. Geng R. -X. Ji Q. (2011). Recent Advances in Researches of Triazole-Based Supramolecular Chemistry and Medicinal Drugs. Chem. J. Chin. Univ. 32, 1970–1985.

[B12] Cortés-García C. J. Chacón-García L. Mejía-Benavides J. E. Díaz-Cervantes E. (2020). Tackling the SARS-CoV-2 Main Protease Using Hybrid Derivatives of 1,5-disubstituted Tetrazole-1,2,3-Triazoles: an In Silico Assay. Peerj Phys. Chem. 2, e10. 10.7717/peerj-pchem.10

[B13] Dalloul H. M. El-nwairy K. Shorafa A. Z. Abu Samaha A. (2017). Synthesis and Biological Activities of Some New spiro 1,2,4-triazole Derivatives Having Sulfonamide Moiety. Org.Commun. 10, 280–287. 10.25135/acg.oc.27.17.08.046

[B14] De Nino A. Algieri V. Tallarida M. A. Costanzo P. Pedrón M. Tejero T. (2019). Regioselective Synthesis of 1,4,5-Trisubstituted-1,2,3-Triazoles from Aryl Azides and Enaminones. Eur. J. Org. Chem. 2019, 5725–5731. 10.1002/ejoc.201900889

[B15] De Nino A. Maiuolo L. Costanzo P. Algieri V. Jiritano A. Olivito F. (2021). Recent Progress in Catalytic Synthesis of 1,2,3-triazoles. Catalysts 11, 1120. 10.3390/catal11091120

[B16] De Nino A. Merino P. Algieri V. Nardi M. Di Gioia M. L. Russo B. (2018). Synthesis of 1,5-functionalized 1,2,3-triazoles Using Ionic liquid/Iron(III) Chloride as an Efficient and Reusable Homogeneous Catalyst. Catalysts 8, 364. 10.3390/catal8090364

[B17] Dhavale D. D. Matin M. M. (2004). Selective Sulfonylation of 4-C-Hydroxymethyl-β-L-Threo-Pento-1,4-Furanose: Synthesis of Bicyclic Diazasugars. Tetrahedron 60 (19), 4275–4281. 10.1016/j.tet.2004.03.034

[B18] El-Reedy A. A. M. Soliman N. K. (2020). Synthesis, Biological Activity and Molecular Modeling Study of Novel 1,2,4-Triazolo[4,3-B][1,2,4,5]tetrazines and 1,2,4-Triazolo[4,3-B][1,2,4]triazines. Sci. Rep. 10, 6137. 10.1038/s41598-020-62977-x 32273529 PMC7145827

[B19] Faria-Ramos I. Tavares P. R. Farinha S. Neves-Maia J. Miranda I. M. Silva R. M. (2014). Environmental Azole Fungicide, Prochloraz, Can Induce Cross-Resistance to Medical Triazoles inCandida Glabrata. FEMS Yeast Res. 14, 1119. 10.1111/1567-1364.12193 25132632

[B20] Farooq T. (2021). Advances in Triazole Chemistry. Amsterdam: Elsevier, 21–27.

[B21] Gao H. Shreeve J. N. M. (2011). Azole-based Energetic Salts. Chem. Rev. 111, 7377–7436. 10.1021/cr200039c 21838230

[B22] Giel M. C. Smedley C. J. Mackie E. R. R. Guo T. Dong J. Soares da Costa T. P. (2020). Metal‐Free Synthesis of Functional 1‐Substituted‐1,2,3‐Triazoles from Ethenesulfonyl Fluoride and Organic Azides. Angew. Chem. Intl Edit 59 (3), 1181–1186. 10.1002/anie.201912728 31709653

[B23] Hahm H. S. Toroitich E. K. Borne A. L. Brulet J. W. Libby A. H. Yuan K. (2020). Global Targeting of Functional Tyrosines Using Sulfur-Triazole Exchange Chemistry. Nat. Chem. Biol. 16, 150–159. 10.1038/s41589-019-0404-5 31768034 PMC6982592

[B24] Hitchcock C. A. Dickinson K. Brown S. B. Evans E. G. V. Adams D. J. (1990). Interaction of Azole Antifungal Antibiotics with Cytochrome P-450-dependent 14α-Sterol Demethylase Purified from Candida Albicans. Biochem. J. 266, 475–480. 10.1042/bj2660475 2180400 PMC1131156

[B25] Huisgen R. (1963). 1,3-Dipolar Cycloadditions. Past and Future. Angew. Chem. Int. Ed. Engl. 2, 565–598. 10.1002/anie.196305651

[B26] Ibraheem S. T. K. Redha W. A. Razak A. Shneshil M. K. (2019). Synthesis of 1,2,3-triazole Derivatives from Azidoacetamide via Cyclo-Addition Reaction. J. Pharm. Sci. Res. 11 (2), 540–544.

[B27] Javaherian M. Kazemi F. Ghaemi M. (2014). A Dicationic, Podand-like, Ionic Liquid Water System Accelerated Copper-Catalyzed Azide-Alkyne Click Reaction. Chin. Chem. Lett. 25, 1643–1647. 10.1016/j.cclet.2014.09.005

[B29] Jørgensen L. N. Matzen N. Heick T. M. Havis N. Holdgate S. Clark B. (2021). Decreasing Azole Sensitivity of Z. Tritici in Europe Contributes to Reduced and Varying Field Efficacy. J. Plant Dis. Prot. 128, 287–301. 10.1007/s41348-020-00372-4

[B30] Kumar S. Khokra S. L. Yadav A. (2021). Triazole Analogues as Potential Pharmacological Agents: a Brief Review. Futur. J. Pharm. Sci. 7, 106. 10.1186/s43094-021-00241-3 34056014 PMC8148872

[B31] Kumari M. Tahlan S. Narasimhan B. Ramasamy K. Lim S. M. Shah S. A. A. (2021). Synthesis and Biological Evaluation of Heterocyclic 1,2,4-triazole Scaffolds as Promising Pharmacological Agents. BMC Chem. 15, 5. 10.1186/s13065-020-00717-y 33478538 PMC7818921

[B32] Kuntala N. Mareddy J. Telu J. R. Banothu V. Pal S. Anireddy J. S. (2021). Nonsteroidal Anti‐inflammatory Drugs Based New 1,2,3‐triazole Derivatives: Their Design, One‐pot Synthesis and *In Vitro* Evaluation. J. Heterocyclic Chem. 58, 2018–2032. 10.1002/jhet.4328

[B33] Lebeau A. Abrioux C. Bénimèlis D. Benfodda Z. Meffre P. (2016). Synthesis of 1,4-disubstituted 1,2,3-triazole Derivatives Using Click Chemistry and Their Src Kinase Activities. Mc 13, 40–48. 10.2174/1573406412666160404125718 27041552

[B34] Li W. Zhou X. Luan Y. Wang J. (2015). Direct Access to 1,4-disubstituted 1,2,3-triazoles through Organocatalytic 1,3-dipolar Cycloaddition Reaction of α,β-unsaturated Esters with Azides. RSC Adv. 5, 88816–88820. 10.1039/C5RA19038J

[B35] Liu X.-G. Zhao X.-L. Zhang Y. Gao J.-R. (2016). An Efficient Three-Component Reaction of Sodium Azide, Haloalkane and Alkyne for the Synthesis of 1, 2, 3-triazoles Catalyzed by the Bifunctional Ionic Liquid Catalyst Choline Chloride-CuCl in Water. Loc 13, 224–230. 10.2174/1570178612666150908213423

[B36] Maiuolo L. Russo B. Algieri V. Nardi M. Di Gioia M. L. Tallarida M. A. (2019). Regioselective Synthesis of 1,5-disubstituted 1,2,3-triazoles by 1,3-dipolar Cycloaddition: Role of Er(OTf)3, Ionic Liquid and Water. Tetrahedron Lett. 60, 672–674. 10.1016/j.tetlet.2019.01.053

[B37] Mao L.-f. Wang Y.-W. Zhao J. Xu G.-Q. Yao X.-J. Li Y.-M. (2020). Discovery of Icotinib-1,2,3-Triazole Derivatives as Ido1 Inhibitors. Front. Pharmacol. 11, 579024. 10.3389/fphar.2020.579024 33101032 PMC7555427

[B38] Matin M. M. Sharma T. Sabharwal S. G. Dhavale D. D. (2005). Synthesis and Evaluation of the Glycosidase Inhibitory Activity of 5-hydroxy Substituted Isofagomine Analogues. Org. Biomol. Chem. 3 (9), 1702–1707. 10.1039/b418283a 15858653

[B39] Mioc M. Avram S. Bercean V. Kurunczi L. Ghiulai R. M. Oprean C. (2018). Design, Synthesis and Biological Activity Evaluation of S-Substituted 1*H*-5-Mercapto-1,2,4-Triazole Derivatives as Antiproliferative Agents in Colorectal Cancer. Front. Chem. 6, 373. 10.3389/fchem.2018.00373 30234098 PMC6134806

[B40] Mioc M. Soica C. Bercean V. Avram S. Balan-Porcarasu M. Coricovac D. (2017). Design, Synthesis and Pharmaco-Toxicological Assessment of 5-Mercapto-1,2,4-Triazole Derivatives with Antibacterial and Antiproliferative Activity. Int. J. Oncol. 50, 1175–1183. 10.3892/ijo.2017.3912 28350123 PMC5363884

[B41] Morozova M. A. Yusubov M. S. Kratochvil B. Eigner V. Bondarev A. A. Yoshimura A. (2017). Regioselective Zn(OAc)2-Catalyzed Azide-Alkyne Cycloaddition in Water: the green Click-Chemistry. Org. Chem. Front. 4, 978–985. 10.1039/C6QO00787B

[B42] Muthipeedika N. J. Yadav D Bodke Y. D. Sandeep Telkar S. Vasily A Bakulev V. A. (2020). Synthesis of Coumarins Linked with 1,2,3-triazoles under Microwave Irradiation and Evaluation of Their Antimicrobial and Antioxidant Activity. J. Mex. Chem. Soc. 64 (1), 53–73. 10.29356/jmcs.v64i1.1116

[B43] Nagarajan R. Jayashankaran J. Emmanuvel L. (2016). Transition Metal-free Steric Controlled One-Pot Synthesis of Highly Substituted N -amino 1,2,3-triazole Derivatives via Diazo Transfer Reaction from β-keto Esters. Tetrahedron Lett. 57, 2612–2615. 10.1016/j.tetlet.2016.04.112

[B44] Odds F. C. Brown A. J. P. Gow N. A. R. (2003). Antifungal Agents: Mechanisms of Action. Trends Microbiol. 11 (6), 272–279. 10.1016/s0966-842x(03)00117-3 12823944

[B45] Phukan P. Kulshrestha A. Kumar A. Chakraborti S. Chattopadhyay P. Sarma D. (2021). Cu(II) Ionic Liquid Promoted Simple and Economical Synthesis of 1,4-Disubstituted-1,2,3-Triazoles with Low Catalyst Loading. J. Chem. Sci. 133, 131. 10.1007/s12039-021-01980-9

[B46] Potts K. T. (1961). The Chemistry of 1,2,4-triazoles. Chem. Rev. 61 (2), 87–127. 10.1021/cr60210a001

[B28] Ram V. Sethi A. Nath M. Pratap R. (2019). “Five-Membered Heterocycles,” in Nomenclature and Chemistry of Three-To-Five Membered Heterocycles (Amsterdam: Elsevier), 149–478. 10.1016/b978-0-08-101033-4.00005-x

[B47] Rondestvedt C. S. Jr. Chang P. K. (1955). Unsaturated Sulfonic Acids. V.1 Addition of Diazomethane and Phenyl Azide to Derivatives of Ethylenesulfonic Acid and its Homologs2. J. Am. Chem. Soc. 77, 6532–6540. 10.1021/ja01629a036

[B48] Rostovtsev V. V. Green L. G. Fokin V. V. Sharpless K. B. (2002). A Stepwise Huisgen Cycloaddition Process: Copper(I)-catalyzed Regioselective "ligation" of Azides and Terminal Alkynes. Angew. Chem. Int. Ed. 41, 2596–2599. 10.1002/1521-3773(20020715)41:14<2596::aid-anie2596>3.0.co;2-4 12203546

[B49] Sagatova A. A. Keniya M. V. Wilson R. K. Sabherwal M. Tyndall J. D. A. Monk B. C. (2016). Triazole Resistance Mediated by Mutations of a Conserved Active Site Tyrosine in Fungal Lanosterol 14α-Demethylase. Sci. Rep. 6, 26213. 10.1038/srep26213 27188873 PMC4870556

[B50] Said M. A. Khan D. J. O. Al-blewi F. F. Al-Kaff N. S. Ali A. A. Rezki N. (2021). New 1,2,3-triazole Scaffold Schiff Bases as Potential Anti-COVID-19: Design, Synthesis, DFT-Molecular Docking, and Cytotoxicity Aspects. Vaccines 9, 1012. 10.3390/vaccines9091012 34579249 PMC8472185

[B51] Seliem I. A. Panda S. S. Girgis A. S. Moatasim Y. Kandeil A. Mostafa A. (2021). New Quinoline-Triazole Conjugates: Synthesis, and Antiviral Properties against SARS-CoV-2. Bioorg. Chem. 114, 105117. 10.1016/j.bioorg.2021.105117 34214752 PMC8219945

[B52] Shafiei M. Peyton L. Hashemzadeh M. Foroumadi A. (2020). History of the development of antifungal azoles: A review on structures, SAR, and mechanism of action. Bioorganic Chemistry 104, 104240. 10.1016/j.bioorg.2020.104240 32906036

[B53] Smedley C. J. Giel M.-C. Molino A. Barrow A. S. Wilson D. J. D. Moses J. E. (2018). 1-Bromoethene-1-sulfonyl fluoride (BESF) is another good connective hub for SuFEx click chemistry. Chem. Commun. 54, 6020–6023. 10.1039/C8CC03400A 29796551

[B54] Surendra Reddy G. Suresh Kumar A. Ramachary D. B. (2020). Organocatalytic enone-azide [3 + 2]-cycloaddition: synthesis of functionally rich C/N-double vinyl 1,2,3-triazoles. Org. Biomol. Chem. 18, 4470–4478. 10.1039/D0OB00848F 32490474

[B55] Taggert B. I. Walker C. Chen D. Wille U. (2021). Substituted 1,2,3-triazoles: a new class of nitrification inhibitors. Sci. Rep. 11, 14980. 10.1038/s41598-021-94306-1 34294800 PMC8298478

[B56] Tao G.-H. Twamley B. Shreeve J. N. M. (2009). A Thermally Stable Nitrogen-Rich Energetic Material-3,4,5-Triamino-1-Tetrazolyl-1,2,4-Triazole (TATT). J. Mater. Chem. 19, 5850–5854. 10.1039/B908214J

[B57] Thomas J. Fokin V. V. (2018). Regioselective Synthesis of Fluorosulfonyl 1,2,3-triazoles from Bromovinylsulfonyl Fluoride. Org. Lett. 20, 3749–3752. 10.1021/acs.orglett.8b01309 29906123

[B58] Thomas J. Jana S. John J. Liekens S. Dehaen W. (2016). A General Metal-free Route towards the Synthesis of 1,2,3-triazoles from Readily Available Primary Amines and Ketones. Chem. Commun. 52, 2885–2888. 10.1039/C5CC08347H 26744743

[B59] Tornøe C. W. Christensen C. Meldal M. (2002). Peptidotriazoles on Solid Phase: [1,2,3]-Triazoles by Regiospecific Copper(I)-catalyzed 1,3-dipolar Cycloadditions of Terminal Alkynes to Azides. J. Org. Chem. 67, 3057–3064. 10.1021/jo011148j 11975567

[B60] Tsogoeva S. Jalani H. Karagöz A. (2017). Synthesis of Substituted 1,2,3-triazoles via Metal-free Click Cycloaddition Reactions and Alternative Cyclization Methods. Synthesis 49, 29–41. 10.1055/s-0036-1588904

[B61] Vaithiyalingam D. Nelson M. Chinnamadhaiyan M. Ayyanar S. (2021). Microwave Assisted Synthesis of 3, 5-Disubstituted 1, 2, 4-Triazole Based Piperazine Amide and Urea Derivatives. Ojc 01, 7–17. 10.31586/ojc.2021.010102

[B62] Woolley D. W. (1944). Some Biological Effects Produced by Benzimidazole and Their Reversal by Purines. J. Biol. Chem. 152 (2), 225–232. 10.1016/S0021-9258(18)72045-0

[B63] Xu H. Ma S. Xu Y. Bian L. Ding T. Fang X. (2015). Copper-catalyzed One-Pot Synthesis of 1,2,4-triazoles from Nitriles and Hydroxylamine. J. Org. Chem. 80 (3), 1789–1794. 10.1021/jo502709t 25564992

[B64] Yang J.-J. Yu W.-W. Hu L.-L. Liu W.-J. Lin X.-H. Wang W. (2020). Discovery and Characterization of 1*H*-1,2,3-Triazole Derivatives as Novel Prostanoid EP4 Receptor Antagonists for Cancer Immunotherapy. J. Med. Chem. 63, 569–590. 10.1021/acs.jmedchem.9b01269 31855426

[B65] Yang Y.-L. Xiang Z.-J. Yang J.-H. Wang W.-J. Xu Z.-C. Xiang R.-L. (2021). Adverse Effects Associated with Currently Commonly Used Antifungal Agents: A Network Meta-Analysis and Systematic Review. Front. Pharmacol. 12, 697330. 10.3389/fphar.2021.697330 34776941 PMC8585744

[B66] Zaher N. H. Mostafa M. I. Altaher A. Y. (2020). Design, Synthesis and Molecular Docking of Novel Triazole Derivatives as Potential CoV Helicase Inhibitors. Acta Pharm. 70 (2), 145–159. 10.2478/acph-2020-0024 31955138

[B67] Zhai Z. Wang Q. Shen Z. Tan C. Weng J. Liu X. (2017). Synthesis and Biological Activity of 1,2,4-triazole Thioether Derivatives Containing Pyrazole Moiety. Chin. J. Org. Chem. 37, 232–236. 10.6023/cjoc201607031

[B68] Zhang H.-Z. Damu G. Cai G.-X. Zhou C.-H. (2014). Current Developments in the Syntheses of 1,2,4-triazole Compounds. Coc 18, 359–406. 10.2174/13852728113179990025

[B69] Zhou X. Xu X. Liu K. Gao H. Wang W. Li W. (2016). Organocatalytic 1,3-Dipolar Cyclo-addition Reaction of β-Keto Amides with Azides - Direct Access to 1,4,5-Trisubstituted 1,2,3-Triazole-4-C-arb-oxamides. Eur. J. Org. Chem. 2016 (10), 1886–1890. 10.1002/ejoc.201600157

[B70] Zonios D. Bennett J. (2008). Update on Azole Antifungals. Semin. Respir. Crit. Care Med. 29 (2), 198–210. 10.1055/s-2008-1063858 18366001

