# Supplementary material for: Triazoles and Their Derivatives: Chemistry, Synthesis, and Therapeutic Applications
Source: Front Mol Biosci. 2022 Apr 25;9:864286. doi: 10.3389/fmolb.2022.864286 (PMC9081720; doi:10.3389/fmolb.2022.864286)
Supplement: Supplementary file 1 [file DataSheet1.docx]

Supplementary Material

Triazoles and Their Derivatives: Chemistry, Synthesis, and Therapeutic Applications

Mohammed M. Matin^*^, Priyanka Matin, Md. Rezaur Rahman, Taibi Ben Hadda, Faisal A. Almalki, Shafi Mahmud, Mohammed M. Ghoneim, Maha Alruwaily, Sultan Alshehri^*^

**Supplementary contents**

1. Figures S1-S7

2. Synthetic method for 1,2,4-triazole analogs

3. Therapeutic application of triazoles

4. Physicochemical properties of some important triazoles

5. References

|   **S1A.** Preparation of 1,4-disubstituted-1,2,3-triazoles. (a) CuI (5 mol%), Na-ascorbate, *^t^*BuOH/H_2_O, rt, 12-72 h; (b) CuSO_4_.5H_2_O (5 mol%), Na-ascorbate (10 mol%), aq. PEG400/H_2_O, rt, 1-8 h.    **S1B.** Synthesis of benzimidazole linked 1,2,3-triazoles.    **S1C.** Synthesis of benzimidazole linked 1,2,3-triazoles.  FIGURE S1 \| Synthetic Schemes for 1,2,3-triazole analogues. |
| --- |

|   **S2A.** Metal-free three-component protocol for the preparation of 1,2,3-triazoles.    **S2B.** Metal-free synthesis of 1-substituted-1,2,3-triazoles.    **S2C.** Metal-free synthesis of 1,4-di-substituted-1,2,3-triazoles.    **S2D.** Organocatalytic (DBU) strategy with β-keto amides forming trisubstituted triazoles.  FIGURE S2 \| Schemes for the synthesis of 1,2,3-triazole analogues. |
| --- |

|   **S3A.** One-pot synthetic strategy for 1-amino-1,2,3-triazoles.    **S3B.** Organocatalytic synthesis of C/N-di-vinyl-1,2,3-triazoles.    **S3C.** IL (ChCl-CuCl) catalyzed three-component cycloaddition reaction.    **S3D.** 1,5-Disubstituted-1,2,3-triazoles synthesis catalyzed by [mPy]OTf/FeCl_3_.    **S3E.** [mPy]OTf catalyzed formation of 1,4,5-trisubstituted-1,2,3-triazoles.  FIGURE S3 \| Synthetic methods for 1,2,3-triazole analogues. |
| --- |

|   **S4A.** [Bmim][CuCl_3_] catalyzed formation of 1,4-disubstituted-triazoles.    **S4B.** MW assisted formation of 1,4-disubstituted-triazoles.    **S4C.** CuIL_1_PPh_3_ catalyzed and US assisted triazole synthesis.  FIGURE S4 \| Synthetic preparation of 1,2,3-triazole analogues. |
| --- |

|   **S5A.** Cu-catalyzed one-pot preparation of 3,5-disubstituted triazoles.    **S5B.** Base-promoted preparation of 3,5-disubstituted-triazoles **46a-c**.    **S5C.** Conversion of 5-mecapto-triazoles to fused triazolo-trizines.  FIGURE S5 \| Synthetic Schemes for 1,2,4-triazole analogues. |
| --- |

|   **S6A.** MW assisted condensation of nitrile with hydrazide to produce 1,2,4-triazoles.    **S6B.** Synthesis of spiro triazoles **53**.    **S6C.** Synthesis of imidazo triazole thioethers **54a-m**.    **S6D.** Synthesis of trisubstituted triazoles **56** and **57**.  FIGURE S6 \| Synthetic methods for 1,2,4-triazole analogues. |
| --- |

|          FIGURE S7 \| Therapeutically important 1,2,3- and 1,2,4-triazole analogues. |
| --- |

# Synthetic method for 1,2,4-triazole analogs

The 1,2,4-triazole-based biologically potential compounds have found enormous applications, which led to the development of different synthetic routes for their preparation. Most of the preparations are based on cyclizations from the necessary starting materials to form the target triazole ring(s). The synthetic routes employed since 2015, so far, are described here under the following categories.

**a) Cu catalyzed synthesis**

Cu-catalyzed stepwise cycloaddition of azides to terminal alkynes i.e. Cu-catalyzed azide-alkyne cycloaddition (CuAAC) is very common in the construction of both the 1,2,3- and 1,2,4-triazoles. Apart from CuAAC, different copper catalysts are used in several reaction conditions such as three-component cyclization reaction, [3+2] cycloaddition reaction, etc. leading to the formation of different substituted triazoles.

Copper-catalyzed three-component reaction among aryldiazonium salts, fluorinated diazo compounds, and nitriles is also reported (Peng et al., 2020). The reaction proceeds via [1+2+2] annulation protocol with *in situ* generations of nitrile ylide with the aryldiazonium salt **S1** under simple conditions (Scheme S1). The scope of this protocol provides a synthesis of divergent drug-like trifluoromethylated *N*^1^-aryl-1,2,4-triazoles **S2**. Later on, the same research group (Zhou et al., 2021) also extended a similar technique to get a wide variety of 1-aryl-5-cyano-1,2,4-triazoles **S3** (**Scheme S1**).

|   **Scheme S1.** Cu-catalyzed three component annulation reactions provided fluoroalkyl-substituted *N*^1^-aryl-1,2,4-triazoles. |
| --- |

In another method, Cu(II) catalyzed [3+2] cycloaddition between aryl diazonium salts **S1** and ethyl cyanoacetate is conducted (Liu et al., 2018). In such a method, the selectivity of the position of substituents is controlled by the choice of catalyst and the yield is high. For example, the use of the Cu(II) catalyst selectively furnished 1,5-disubstituted-1,2,4-triazoles **S4**, whereas the use of the Ag(I) catalyst provided 1,3-disubstituted-1,2,4-triazoles **S5** (**Scheme S2**).

|   **Scheme S2**. Highly regioselective substituted synthesis of 1,2,4-triazoles. |
| --- |

Cu(II) catalyzed C–N bond-forming ability over C–S was utilized arylidene-aryl thiosemicarbazides **S6** to prepare 4,5-disubstituted-1,2,4-triazole-3-thiones **S7** (**Scheme S3**). In this method, the *in situ* formed thiones are transformed into **S7** via desulfonation after a longer reaction time (Gogoi et al., 2015). The **S7** compounds possess antimicrobial potentiality.

|   **Scheme S3**. Cu(II) catalyzed oxidative-heterocyclization for the synthesis of 1,2,4-triazoles. |
| --- |

Heterogeneous copper(I)-catalyzed practical and efficient technique for the preparation of 1,2,4-triazoles are described by Xia et al. (2019). They used a recyclable and inexpensive heterogeneous catalyst namely 1,10-phenanthroline-functionalized MCM-41-supported copper(I) complex [Phen-MCM-41-CuBr] (MCM-41 = Mobil Composition of Matter No. 41). Thus, nitriles underwent addition-oxidative cyclization with amidines (2-aminopyridines) **S8** in DMSO or 1,2-dichlorobenzene at 120–130 °C employing Phen-MCM-41-CuBr catalyst and normal air oxidant (**Scheme S4**) to yield 1,2,4-triazole derivatives **S9** in acceptable yields.

|   **Scheme S4**. Heterogeneous Cu(I) catalyzed addition-oxidative cyclization for the preparation of 1,2,4-triazoles. |
| --- |

Very recently, Zhang et al. (2022) developed Cu-catalyzed decarbonylative cyclization technique to get fluorinated and trisubstituted triazole **S12** from isatins **S10** and trifluoroacetimidohydrazides **S11** (**Scheme S5**). The synthesis proceeds via a cascade condensation followed by hydrolysis, decarboxylation, and intramolecular C−N bond formation. More advantageously, the products **S12** with an amino group can further be modified to incorporate pharmacophoric groups.

|   **Scheme S5.** Cu-catalyzed three component annulation reactions provided fluoroalkyl-substituted *N*^1^-aryl-1,2,4-triazoles **S12**. |
| --- |

**b) Base catalyzed synthesis**

Sonawane et al. (2017) reported the preparation of 1,2,4-triazole-3-thione analogues **S14** using base (KOH/EtOH) catalyzed cyclization of aroyldithiocarbazate **S13** (**Scheme S6**).

|   **Scheme S6.** Base-catalyzed cyclization of aroyldithiocarbazate. |
| --- |

A weak organic base like triethylamine (TEA) is also used as a catalyst in the cyclization process to generate 1,2,4-triazoles (Aly et al., 2019). Thus, the treatment of amidrazones **S15** with ethyl azodicarboxylate in the presence of TEA (triethylamine, Mitsunobu reagent) in EtOH formed regioselective 1,3,5-trisubstituted-1,2,4-triazoles **S16** (**Scheme S7**).

|   **Scheme S7.** Regioselective formation of 1,2,4-triazoles catalyzed by TEA. |
| --- |

**c) MW-assisted method**

There are very few microwave-assisted methods available for the synthesis of 1,2,4-triazoles. We have already discussed one such method in the main article.

Microwave-assisted catalyst-free, mild, efficient, and very simple conditions for the preparation of 1-substituted-1,2,4-triazoles are reported (Shelke et al., 2015). MW irradiation at 160 °C between hydrazines and excess formamide in absence of catalyst furnished 1,2,4-triazole **S17** in 54-81% yields (**Scheme S8**).

|   **Scheme S8.** MW-assisted preparation of 1-substituted-1,2,4-triazoles **S17**. |
| --- |

In addition, environmentally benign microwave (MW) conditions were applied for the derivatization of ester triazoles to amide triazoles (Jaisankar et al., 2015). Initially, cyclization of amine **S18** with phenylacetyl chloride under reflux in toluene gave triazole **S19** (**Scheme S9**). Compound **S19** was then converted into triazole amides **S20**.

|   **Scheme S9.** MW-assisted preparation of 1,2,4-triazole-3-carboxamides **S20**. |
| --- |

**d) Miscellaneous methods**

In addition to the previously mentioned methods, several other cyclization conditions for the synthesis of 1,2,4-triazoles are reported. Many metal-free conditions are reported which led to the formation of substituted 1,2,4-triazoles. For example, aerobic oxidative conditions for C–H functionalization followed by double C–N bonds formation, and oxidative aromatization were achieved by treating with molecular iodine and *t*-butylhydroperoxide (TBHP) of a mixture of hydrazones **S21** and aliphatic amines **S22** (Chen et al., 2016) and ultimately produced 1,3,5-trisubstituted-1,2,4-triazoles **S23** (**Scheme S10**).

|   **Scheme S10.** Metal-free I_2_-catalyzed oxidative coupling between of hydrazones and amines. |
| --- |

In a similar style, I_2_ catalyzed oxidative C-N and N-S bond formations in aqueous media are developed (Jatangi et al., 2018). As shown in **Scheme S11**, a mixture of *N*-arylbenzamidrazone **S24** and phenyl isothiocyanates **S25** on treatment with molecular iodine (oxidant) in H_2_O furnished 4,5-disubstituted-3-amino-1,2,4-triazoles **S26**. This facile method showed several advantages like high product yields, substrate tolerance, and being environmentally benign.

|   **Scheme S11.** I_2_-Catalyzed synthesis of 4,5-disubstituted-3-amino-1,2,4-triazoles **S26**. |
| --- |

A novel electrochemical one-pot multicomponent reaction method for substituted 1,2,4-triazole synthesis is reported by Yang and Yuan (2018). In recent years, synthetic organic electrochemistry is getting more interest. Of the electrochemical techniques, the reactive iodide radical or iodine electrogenerated *in situ* at an anode can effectively catalyze some transformations. Thus, the multicomponent treatment/reaction among aryl hydrazines **S27**, paraformaldehyde, ammonium acetate, and alcohols under electrolysis in a cell constructed with a graphite rod anode and a Ni plate cathode mediated by TBAI and in presence of base KO*^t^*Bu furnished 1,5-disubstituted-1,2,4-triazoles **S28** (**Scheme S12**). This method advantageously avoids the use of transition-metal catalysts and hazardous oxidant reagents.

|   **Scheme S12.** Electrochemical route for the synthesis of 1,5-disubstituted-1,2,4-triazoles **S28**. |
| --- |

# Therapeutic application of triazoles

Triazole-based many drugs (such as fluconazole, triazolam, alprazolam, Rufinamide, itraconazole, lamtidine, ribavirin, rassinazole, and sitagliptin) are already in clinical treatment. The excellent aromatic character and richness of electrons of triazole structures enable them to bind with many types of enzymes and receptors. They are able to bind with weak interactions like coordination bonds, hydrogen bonds, ion-dipole, hydrophobic effect, pi-pi interaction, non-bond interaction, and van der Waals forces. Due to multidrug resistance in several cases, new and efficient drugs are extremely essential. Thus, a plethora of triazole derivatives have been designed and synthesized in the last couple of years. Investigation of the pharmacological effects (pharmacophore, binding interactions with enzyme, ADMET, etc.) of new triazole derivatives helped to develop novel drugs.

Brief biological and therapeutic applications of some newly synthesized triazoles are presented below.

# a) Antimicrobial agents

# Triazoles like fluconazole, itraconazole, voriconazole, and posaconazole (Figure 1) are used in the therapy of *Candida* infections. Many substituted triazoles are reported to show inhibition against drug-resistant microorganisms, which disrupts the eﬀectiveness of the available drugs. For example, isavuconazole, the newest triazole, is active against broad-spectrum organisms including *Mucorales* and dimorphic fungi. It is an alternative first-line drug for the treatment of pulmonary aspergillosis (Miceli and Kauffman, 2015).

# Ellouz et al. (2018) synthesized and evaluated antibacterial activity *in vitro* of 1,2,3-triazolylmethyl-2*H*-1,4-benzothiazin-3(4*H*)-one analogue where compound S29 exhibited promising activities against *Pseudomonas aeruginosa* ATCC 27853 and *Acinetobacter* ESBL (MIC= 31.2 μg/mL) (Ellouz et al., 2018). Some triazole-thiazole hybrids S30 are found to possess promising inhibitory activity against the bacterial strains (IC_50_ = 2.8 to 15.7 µM) (Gondru et al., 2021). Especially these compounds are highly active against *B. subtilis*, *Candida strains*, and *S. aureus*.

|  |
| --- |

Several novel S-substituted 4-alkyl-5-((3-(pyridin-4-yl)-1*H*-1,2,4-triazole-5-yl)thio)methyl)-4*H*-1,2,4-triazole-3-thiol are synthesized and antimicrobial activities are tested (Karpun et al., 2021). Encouragingly, synthesized triazole **S31** exhibited strong sensitivity against four pathogenic strains (*S. fecalis*, *S. pullorum*, *S. typhimurium*, and *K. pneumonia*) with very low MBC (15.6 µg/mL).

|  |
| --- |

# b) Anti-tubercular triazoles

The causative agent of Tuberculosis (TB) is a pathogenic bacteria named *Mycobacterium tuberculosis* (MTB). This contagious and infectious disease emerges as the deadliest disease. Both the 1,2,3- and 1,2,4-triazole derivatives have shown potentiality against TB both *in vitro* and *in vivo*, and are considered an effective anti-TB agents. The most important triazoles with anti-TB activities are discussed here along with the structure-activity relationship (SAR).

Three series 1,2,3-triazole-based quinoxaline-1,4-di-*N*-oxide derivatives were synthesized and tested anti-tubercular activities *in vitro* against H37Rv strain isolated from clinical samples (Srinivasarao et al., 2020). Most of the compounds are active against this strain and the SAR study revealed that compound **S32** is the best active against TB with MIC 30.35 µM and less kidney toxicity.

|  |
| --- |

Instead of quinoxaline-1,4-di-*N*-oxide, phthalimide bearing 1,2,3-triazoles **S33a-b** are prepared and are found very effective against *M. tuberculosis* (MTB) H37Rv (MIC 12.5 µg/mL) (Phatak et al., 2019). It is also reported that 1,2,3-triazole **S34** linking saccharin and usnic acid moieties is a highly active MTB agent with MIC 2.5 μM (Bangalore et al., 2020).

|  |
| --- |

The anti-tubercular activity of pyridin-4-yl-pyrimido-triazolo-triazine-6-carboxylic acid **S35a-g** against bacterial strain MTB H37Rv ATCC by microplate alamar blue assay method indicated that all the compounds are sensitive at 50 μg/mL concentration (Babu et al., 2020). However, triazoles with R = phenyl, 2-nitrophenyl, and 4-fluorophenyl substituents are active against MTB at very low concentrations (6.25µg/mL). All these results indicated that the triazole derivatives have attracted great interest in searching for novel anti-MTB agents.

|  |
| --- |

# c) Anticancer therapy

# Anticancer drug (oncology field) discovery is a top priority in medical science. Triazole scaffolds with different substituents (pharmacophores) are being widely synthesized and extensively tested. Many of them showed significant antiproliferative activities in cancer treatments both *in vitro* and *in vivo*.

Recently, a novel series of 1,2,3-triazole hybrids based on myrrhanone B (28 compounds) were synthesized, and their antiproliferative potentiality against several cell lines (A549, DU145, MDA-MB-231, SiHa, U87MG, PC-3, HT-29, etc.) was assessed (Madasu et al., 2020). Compound **S36a** (IC_50_: 6.57±0.62 µM) and **S36b** (IC_50_: 10.85±0.90 µM) showed almost identical inhibitory properties with the standard anticancer drug doxorubicin (IC_50_: 5.05±0.25 µM).

|  |
| --- |

Chromene containing 1,2,3-triazoles **S37** have also shown broad-spectrum activity against six human cancer cell lines (Luan et al., 2020). The structure-activity relationship study indicated that the fluoro atom joined to the aromatic (phenyl) ring mostly added the efficacy against the A549 lung cancer cell line (IC_50_: 1.02-74.27 μm, an MTT assay). In addition, 1,2,3-triazole hybrids **S38-41** possess broad-spectrum anticancer activity (IC_50_ values at nM scale) (Irfan et al., 2016; Amdouni et al., 2017).

|    |
| --- |

# As described in the main manuscript, many *S*-substituted 3-aryl-5-mercapto-1,2,4-triazoles 46a-c (Figure S5b) are highly active in the HT-29 cancer cell line. Again, using MTT assay El-Sherief et al. (2018) showed that 1,2,4-triazoles S42 have remarkable antiproliferative potentiality against a panel of cancer cell lines including EGFR, BRAF, and Tubulin anticancer targets.

Maddali et al. (2021) synthesized several novel 4,5-diphenyloxazol-1,2,4-triazole derivatives **S43a-c** and conducted their anticancer activities against prostate lung cancer cell lines viz., PC-93 and HBT-55. Encouragingly, **S43a-c** showed excellent antiproliferative activity against the HBT-55 cell line (IC_50_ = 17.28, 16.48, and 15.12 μM respectively) and comparable to the doxorubicin.

|   |
| --- |

In general, during anticancer activity, the triazole unit acts as a linker between two anticancer pharmacophores or hybridizes with other pharmacophores of anticancer potential.

**d) Anti-inflammatory agents**

Angajala et al. (2016) synthesized several 1,2,3-triazoles **S44a-q** linked to the ibuprofen unit via an aromatic linker. Anti-inflammatory (AI) activities (*in vivo*) of all 1,2,3-triazoles indicated that **S44a** possesses better efficacy than ibuprofen (reference AI drug). Compound **S44a** might be used as a nonsteroidal anti-inflammatory drug (NSAID) in analgesic, antipyretic, rheumatic arthritis therapy.

|  |
| --- |

Novel aryl-substituted 1,2,4-triazole compounds **S45,** **S46a,b,** and **S47** are reported as potential/excellent anti-inflammatory agents (Al-Turki et al., 2015; Khan et al., 2018). **S45** is found selective cyclooxygenase-2 (COX-2) inhibitor at low concentration compared to the standard drug celecoxib (Al-Turki et al., 2015). Also, compound **S46a,b** exhibited antiproliferative activity and diverse effects on cytokine production with very low toxicity (Paprocka et al., 2015).

|  |
| --- |

**e) Antiviral treatment**

In search of triazole-based effective viral therapy resulted in the establishment of several antiviral drugs. In this regard, sugar moieties and substituted pyridines linked by 1,2,3-triazoles are synthesized, and subjected for MTT cytotoxicity assay against H5N1 influenza virus (El-Sayed et al., 2017). Of the novel triazole conjugates compound **S48** exhibited high activity with low toxicity.

Again, ribavirin (RBV) having broad-spectrum antiviral activities are found to inhibit coronaviruses related in Vero and LLC-MK2 cells, although at high concentrations (Falzarano et al., 2013). However, RBV in combination with interferon-α2b is now in clinical use and might be a drug of choice against COVID-19 infections (Hung et al., 2020).

|  |
| --- |

In an aim to interactions at the hydrophobic channel of NNIBP, Zhou et al. (2019) discovered three novel diarylpyrimidine-based 1,2,3-triazoles **S49a-c** with superior activity against wild-type and K103N mutant virus. These triazoles have higher potency and lower cytotoxicity compared to the first-line antiretroviral HIV drug efavirenz.

|  |
| --- |

**f) Trizoles as antioxidants**

Incorporation of 1,2,3-triazoles with starch (e.g. 6-hydroxyethyltriazole-6-deoxy starch, 6-hydroxymethyltriazole-6-deoxy starch, 6-hydroxypropyltriazole-6-deoxy starch, etc.) increased its antioxidant activity significantly (Tan et al., 2016). Also, the electron-donating capability of various substituted groups of the triazole ring increases their antioxidant property. Chromene-based 1,2,3-triazole derivatives are reported to possess strong scavenging properties (Ihnatova et al., 2021). In addition, 1,2,4-triazole derivatives are found to exhibit a stabilizing effect on cell membranes. Also, these compounds displayed antioxidant activity with respect to erythrocytes under oxidative stress related-conditions (Kochikyan et al., 2011).

**g) Other therapeutic applications**

More therapeutic applications of triazole derivatives are uncovered in recent years. For example, Deferasirox (DFX), a trisubstituted-1,2,4-triazole, is the newest oral iron chelators (binder) available worldwide to treat chronic iron overload in iron-loading anemia (Piolatto et al., 2021). However, DFX activity can be further enhanced with the intake of vitamin E, and omeprazole (Hamed et al., 2020).

Several *S*-substituted 1,2,4-triazol-3-thiols derivatives **S50a,b** was found to be potent inhibitors of α-glucosidase enzyme and even better than acarbose (standard α-glucosidase inhibitor for type II diabetes) (Ur-Rehman et al., 2018). The low hemolytic activity of these triazoles indicated their prospect as new antidiabetic drug candidates.

|  |
| --- |

# Physicochemical properties of some important triazoles

To rationalize the promising biological/therapeutic actions of triazole compounds several researchers reported their physicochemical properties (ADMET, binding sites, etc.) in addition to synthetic techniques (Nehra et al., 2021).

The most widely used triazole, fluconazole has excellent oral absorption, high penetration in the cerebrospinal and ocular fluids, and higher renal excretion. Advantageously, it isn’t affected by food consumption. Among the physicochemical properties, the water solubility of the drug molecules is very important and related to their bioactivities and application. Due to the superior potency of **S49a-c**, Zhou et al. (2019) reported their physicochemical properties (especially drug-likeness properties) and water solubility. All these compounds **S49a-c** have good water solubility in different pH levels due to the presence of triazole unit. Also, the other physicochemical parameters related to drug-likeness (MW, HBD, HBA, Log*P*o/w) permits Lipinski’s rule-of-five. In addition, their topological polar surface area (TPSA) is found below 127 Å^2^ (<140 Å^2^), and hence should have better intestinal absorption with reduced central nervous toxicity.

As shown in Scheme 22 (original article), the promising antimicrobial and anticancer agents **57** (20 compounds) are solid (mp 121-214 °C) and mostly colored (Kumari et al., 2021). According to their SAR study, biological activities are dependent on aryl substituents. For example, the presence of CHO group at *p*-position increased antifungal, trimethoxy groups at *m*,*p*-positions enhanced antibacterial, and *o*-OH and *p*-CHO increased antiproliferative (anticancer) properties.

|  |
| --- |

# References

Al-Turki, D. A., Al-Omar, M. A., Abou-Zeid, L. A., Shehata, I. A., and Al-Awady, M. S. (2015). Design, synthesis, molecular modeling and biological evaluation of novel diaryl heterocyclic analogs as potential selective cyclooxygenase-2 (COX-2) inhibitors. *Saudi Pharm. J.* 25, 59–69. doi: 10.1016/j.jsps.2015.07.001

Aly, A. A., Hassan, A. A., Mohamed, N. K., Ramadadfon, M., Brown, A. B., Abd El-Aal, A. S., et al., (2019). Regioselective formation of 1,2,4-triazoles by the reaction of amidrazones in the presence of diethyl azodicarboxylate and catalyzed by triethylamine. *Mol. Divers.* 23, 195–203. doi: 10.1007/s11030-018-9868-6

Amdouni, H., Robert, G., Driowya, M., Furstoss, N., Metier, C., Dubois, A., et al. (2017). In vitro and in vivo evaluation of fully substituted (5-(3-ethoxy-3-oxopropynyl)-4-(ethoxycarbonyl)-1,2,3-triazolyl-glycosides as original nucleoside analogs to circumvent resistance in myeloid malignancies. *J. Med. Chem.* 60, 1523-1533. doi: 10.1021/acs.jmedchem.6b01803

Angajala, K. K., Vianala, S., Macha, R., Raghavender, M., Thupurani, M. K., and Pathi, P. J. (2016). Synthesis, anti-inflammatory, bactericidal activities and docking studies of novel 1,2,3-triazoles derived from ibuprofen using click chemistry. *SpringerPlus* 5:423. doi: 10.1186/s40064-016-2052-5

Babu, N. R., Kulandaivelu, U., Rao, G. S. N. K., Alavala, R. R., Padmavathi, Y., and Reddy, B. M. (2020). Design, synthesis and evaluation of anti tubercular activity of novel triazole derivatives. *Int. J. Pharm. Sci. Res.* 11(6), 2920-2925.

Bangalore, P. K., Vagolu, S. K., Bollikanda, R. K., Veeragoni, D. K., Choudante, P. C., Misra, S., et al. (2020). Usnic acid enaminone-coupled 1,2,3-triazoles as antibacterial and antitubercular agents. *J. Nat. Prod.* 83(1), 26-35. doi: 10.1021/acs.jnatprod.9b00475

Chen, Z., Li, H., Dong, W., Miao, M., and Ren, H. (2016). I_2_-Catalyzed oxidative coupling reactions of hydrazones and amines and the application in the synthesis of 1,3,5-trisubstituted 1,2,4-triazoles. *Org. Lett.*  18(6), 1334-1337. doi: 10.1021/acs.orglett.6b00277

Ellouz, M., Sebbar, N. K., Fichtali, I., Ouzidan, Y., Mennane, Z., Charof, R., et al. (2018). Synthesis and antibacterial activity of new 1,2,3-triazolylmethyl-2*H*-1,4-benzothiazin-3(4*H*)-one derivatives. *Chem****.*** *Central J****.*** 12:123. doi: 10.1186/s13065-018-0494-2

El-Sayed, W. A., Khalaf, H. S., Mohamed, S. F., Hussien, H. A., Kutkat, O. M., and Amret, A. E. (2017). Synthesis and antiviral activity of 1,2,3-triazole glycosides based substituted pyridine via click cycloaddition. *Russ. J. Gen. Chem.* 87, 2444-2453. doi: 10.1134/S10703632171002

El-Sherief, H. A. M., Youssif, B. G. M., Bukhari, S. N. A, Abdel-Aziz, M., Abdel-Rahman, H. M. (2018). Novel 1,2,4-triazole derivatives as potential anticancer agents: Design, synthesis, molecular docking and mechanistic studies. *Bioorg. Chem.* 76:314-325. doi: 10.1016/j.bioorg.2017.12.013

Falzarano, D., De Wit, E., Martellaro, C., Callison, J., Munster, V. J., Feldmann, H. (2020). Inhibition of novel β coronavirus replication by a combination of interferon-α2b and ribavirin. *Sci. Rep.* 3:1686. doi: 10.1038/srep01686

Gogoi, A., Guin, S., Rajamanickam, S., Rout, S. K., and Patel, B. K. (2015). Synthesis of 1,2,4-triazoles via oxidative heterocyclization: Selective C–N bond over C–S bond formation. *J. Org. Chem.* 80(18), 9016–9027. doi: 10.1021/acs.joc.5b00956

Gondru, R., Kanugala, S., Raj, S., Kumar, C. G., Pasupuleti, M., Banothu, J., et al. (2021). 1,2,3-Triazole-thiazole hybrids: Synthesis, in vitro antimicrobial activity and antibiofilm studies. *Bioorg. Med. Chem. Lett.* 33:127746. doi: 10.1016/j.bmcl.2020.127746

Hamed, E. M., Meabed, M. H., Hussein, R. R. S., and Aly, U. F. (2020). Recent insight on improving the iron chelation efficacy of deferasirox by adjuvant therapy in transfusion dependent beta thalassemia children with sluggish response. *Expert Opin. Drug Metab. Toxicol.* 16(3), 179-193. doi: 10.1080/17425255.2020.1729353

Hung, I. F. N., Lung, K. C., Tso, E. Y. K., Raymond Liu, R., Chung, T. W. –H., Chu, M. -Y., et al. (2020). Triple combination of interferon beta-1b, lopinavir–ritonavir, and ribavirin in the treatment of patients admitted to hospital with COVID-19: an open-label, randomised, phase 2 trial. *Lancet* 395(10238), 1695–1704. doi: 10.1016/S0140-6736(20)31042-4

Ihnatova, T., Kaplaushenko, A., Frolova, Y., and Pryhlo, E. (2021). Synthesis and antioxidant properties of some new 5-phenethyl-3-thio-1,2,4-triazoles. *Pharmacia*, *68*, 129-133.

Irfan, M., Khan, S. I,. Manzoor, N., and Abid, M. (2016). Biological activities and in silico physico-chemical properties of 1,2,3-triazoles derived from natural bioactive alcohols. *Anti-Infect. Agents* 14, 126-132. doi: 10.2174/2211352514666160606115200

Jaisankar, K. R., Kumaran, K., Kamil, S. R. M., and Srinivasan, T. (2015). Microwave-assisted synthesis of 1,2,4-triazole-3-carboxamides from esters and amines under neutral conditions. *Res. Chem. Intermed.* 41, 1975–1984. doi: 10.1007/s11164-013-1325-7

Jatangi, N., Tumula, N., Palakodety, R. K., and Nakka, M. (2018). I2-Mediated oxidative C–N and N–S bond formation in water: A metal-free synthesis of 4,5-disubstituted/N-fused 3-amino-1,2,4-triazoles and 3-substituted 5-amino-1,2,4-thiadiazoles. *J. Org. Chem.* 83, 5715-5723. doi: 10.1021/acs.joc.8b00753

Karpun, Y., Parchenko, V., Fotina, T., Demianenko, D., Fotin, A., Nahornyi, V., et al. (2021). The investigation of antimicrobial activity of some s-substituted bis-1,2,4-triazole-3-thiones. *Pharmacia* 68(4), 797–804. Doi: 10.3897/pharmacia.68.e65761

Khan, S., Imam, S., Ahmad, A., Basha, S., and Husain, A. (2018). Synthesis, molecular docking with COX 1& II enzyme, ADMET screening and in vivo anti-inflammatory activity of oxadiazole, thiadiazole and triazole analogs of felbinac. *J. Saudi Chem. Soc.* 22(4), 469-484. doi: 10.1016/j.jscs.2017.05.006

Kochikyan, T. V., Samvelyan, M. A., Arutyunyan, E. V., Arutyunyan, V. S., Avetisyan, A. A., Malakyan, M. G., et al. (2011). Synthesis and antioxidant activity of new 1,2,4-triazole derivatives. *Pharm. Chem. J.* 44, 525–527. doi: 10.1007/s11094-011-0509-y

Liu, J. -Q., Shen, X., Wang, Y., Wang, X.-S., and Bi, X. (2018). [3 + 2] Cycloaddition of csocyanides with aryl diazonium salts: Catalyst-dependent regioselective synthesis of 1,3- and 1,5-disubstituted 1,2,4-triazoles. *Org. Lett. 20*(21), 6930-6933. doi: 10.1021/acs.orglett.8b03069

Luan, T., Quan, Z., Fang, Y., and Yang, H. (2020). Design, Synthesis and Antiproliferative Activity of Chrysin Derivatives Bearing Triazole Moieties. *Chin. J. Org. Chem*. 40, 440–446. doi:10.6023/cjoc201907012

Madasu, C., Shailaja, K., Sangaraju, R., Sistla, R., and Uppuluri, V. M. (2020). Synthesis and biological evaluation of some novel 1,2,3-triazole hybrids of myrrhanone B isolated from Commiphora mukul gum resin: Identification of potent antiproliferative leads active against prostate cancer cells (PC-3). *Eur. J. Med. Chem.* 188:111974. doi: 10.1016/j.ejmech.2019.111974

Maddali, N. K., Ivaturi, V. K. V., Yellajyosula, L. N. M., Malkhed, V., Brahman, P. K., Pindiprolu, S. K. S., (2021). New 1,2,4-triazole scaffolds as anticancer agents: Synthesis, biological evaluation and docking studies. *ChemistrySelect* 6, 6788-6796. doi: 10.1002/slct.202101387

# Miceli, M. H., and Kauffman, C. A. (2015). Isavuconazole: A new broad-spectrum triazole antifungal Agent. *Clin. Infect. Dis.* 61(10), 1558-1565. doi: 10.1093/cid/civ571

Nehra, N., Tittal, R. K., and Ghule, V. D. (2021). 1,2,3-Triazoles of 8‑hydroxyquinoline and HBT: Synthesis and studies (DNA binding, antimicrobial, molecular docking, ADME, and DFT). *ACS Omega* 6, 27089-27100. doi: 10.1021/acsomega.1c03668

Paprocka, R., Wiese, M., Eljaszewicz, A., Helmin-Basa, A., Gzella, A., Modzelewska-Banachiewicz, B., et al., (2015). Synthesis and anti-inflammatory activity of new 1,2,4-triazole derivatives. *Bioorg. Med. Chem. Lett.* 25, 2664–2667. doi: 10.1016/j.bmcl.2015.04.079

Peng, X., Zhang, F. -G., and Ma, J. -A. (2020). Cu-Catalysed three-component reaction of aryldiazonium salts with fluorinated diazo reagents and nitriles: Access to difluoroand trifluoromethylated N1-aryl-1,2,4-triazoles. *Adv. Synth. Catal.* 362(20), 4432-4437. doi: 10.1002/adsc.202000776

Phatak, P. S., Bakale, R. D., Dhumal, S. T., Dahiwade, L. K., Choudhari, P. B., Krishna, V. S., et al. (2019). Synthesis, antitubercular evaluation and molecular docking studies of phthalimide bearing 1,2,3-triazoles. *Syn. Commun*. 49(16), 2017-2028. doi: 10.1080/00397911.2019.1614630

Piolatto, A., Berchialla, P., Allegra, S., Francia, S. D., Ferrero, G. B., Piga, A., et al. (2021). Pharmacological and clinical evaluation of deferasirox formulations for treatment tailoring. *Sci. Rep.* 11:12581. doi: 10.1038/s41598-021-91983-w

Shelke, G. M., Rao, V. K., Jha, M., Cameron, T. S., and Kumar, A. (2015). Microwave-assisted catalyst-free synthesis of substituted 1,2,4-triazoles. *Synlett* 26(3), 404-407. doi: 10.1055/s-0034-1379734

Sonawane, A. D., Rode, N. D., Nawale, L., Joshi, R. R., Joshi, R. A., Likhite, A. P., et al., (2017). Synthesis and biological evaluation of 1,2,4-triazole-3-thione and 1,3,4-oxadiazole-2-thione as antimycobacterial agents. *Chem. Biol. Drug Des.* 90(2), 200–209. doi: 10.1111/cbdd.12939

Srinivasarao, S., Nandikolla, A., Suresh, A., Ewa, A. -K., Głogowska, A., Ghosh, B., et al. (2020). Discovery of 1,2,3-triazole based quinoxaline-1,4-di-N-oxide derivatives as potential anti-tubercular agents. *Bioorg. Chem.* 100: 103955 doi: 10.1016/j.bioorg.2020.103955

Tan, W., Li, Q., Li, W., Dong, F., and Guo, Z. (2016). Synthesis and antioxidant property of novel 1,2,3-triazole-linked starch derivatives via ‘click chemistry’. *Int. J. Biol. Macro.* 82, 404-410. doi: 10.1016/j.ijbiomac.2015.10.007

Ur-Rehman, A., Nafeesa, K., Abbasi, M. A., Siddiqu, S. Z., Rasool, S., Adnan Ali Shah, S. A. A., et al. (2018). S-substituted derivatives of 1,2,4-triazol-3-thiol as new drug candidates for type II diabetes. *Turk. J. Chem.* 42(3), 652-67. doi: 10.3906/kim-1705-17

Xia, J., Huang, X., and Cai, M. (2019). Heterogeneous copper(I)-catalyzed cascade addition–oxidative cyclization of nitriles with 2-aminopyridines or amidines: Efficient and practical synthesis of 1,2,4-triazoles. *Synthesis* 51, 2014-2022. doi: 10.1055/s-0037-1611712

Yang, N., and Yuan, G. (2018). A multicomponent electrosynthesis of 1,5-disubstituted and 1-aryl 1,2,4-triazoles. *J. Org. Chem.* 83(19), 11963-11969. doi: 10.1021/acs.joc.8b01808

Zhang, Y., Yang, Z., Chen, Z., Liu, L., and Wu, X. -F. (2022). Copper-catalyzed decarbonylative cyclization of isatins and trifluoroacetimidohydrazides for the synthesis of 2-(5-trifluoromethyl-1,2,4-triazol-3-yl)anilines. *Adv. Synth. Catal.* doi: 10.1002/adsc.202101397

Zhou, X. Z., Liu, T., Wu, G., Kang, D., Fu, Z., Wang, Z. et al. (2019). Targeting the hydrophobic channel of NNIBP: Discovery of novel 1,2,3-triazole-derived diarylpyrimidines as novel HIV-1 NNRTIs with high potency against wild-type and K103N mutant virus. Org. Biomol. Chem. 17(12), 3202-3217. doi: 10.1039/C9OB00032A
